# Seemingly trivial lesion with devastating consequences

**DOI:** 10.1002/jha2.581

**Published:** 2022-09-27

**Authors:** Edwin Uriel Suárez M., Paula Villalba‐Cuesta, Teresa Arquero, Pilar Llamas, Laura Solán

**Affiliations:** ^1^ Department of Hematology Hospital Universitario Fundación Jiménez Díaz Madrid Spain; ^2^ Department of Medical Oncology Hospital Universitario Fundación Jiménez Díaz Madrid Spain

1

Dear Editor, a 70‐year‐old man presented to the emergency department with pain in his right lower extremity. His relevant past medical history included myelodysplastic syndrome (MDS), multilineage dysplasia subtype, initially categorized as low risk (International Prognostic Scoring System Revised version). Active treatment was not indicated. During a follow‐up consultation 4 years later, myeloblasts were described for the first time in peripheral blood (<5%) with hemoglobin (Hb) of 12 g/dl, a white blood count (WBC) of 7 × 10^3^/µl, and a platelet count of 200 × 10^3^/µl. A new bone marrow evaluation showed grade 3 myelofibrosis. The treatment proposed was 75 mg/m^2^ of subcutaneous azacitidine for 7 days, every 4 weeks. Twenty four hours after finishing his first cycle, the patient presented with induration, erythema, and progressive pain in the right lower extremity coinciding with the sites of subcutaneous administration. He developed fever ∼48 h after the last dose of azacitidine.

On arrival at the emergency room, he had fever of 39°C, but his other vital signs were normal. Skin examination revealed a lesion in the right thigh that measured ∼5 cm, which was tender, erythematous, with purple areas and a blistered surface (Figure 1, panels A–D). The skin surrounding the lesion felt indurated and swollen. No skin crepitus was noted, and distal pulses were intact. An erythematous painless lesion was observed in the contralateral leg, with a scaling of the skin, which also corresponded to another azacitidine application (Figure 1, panel E). Laboratory blood studies revealed the following: Hb 8 g/dl, WBC 12 × 10^3^/µl, absolute neutrophil count 10 × 10^3^/µl, platelets 27 × 10^3^/µl, C‐reactive protein 29 mg/dl, and procalcitonin 5.7 ng/ml. The results of all other blood tests were unremarkable. On admission, empiric antibiotic therapy was started with piperacillin–tazobactam. On the second day of hospitalization, he presented arterial hypotension and persistent fever, and antibiotic therapy was escalated to meropenem and daptomycin. A magnetic resonance imaging (MRI) of the upper leg was performed, showing signs of fasciitis of the right thigh (Figure 2). A skin biopsy was carried out and showed an acute inflammatory infiltrate of polymorphonuclear neutrophils affecting the entire thickness of the skin from the epidermis to the fascia, with necrosis, vessels with fibrin thrombi, and signs of vasculitis, compatible with necrotizing fasciitis (NF). The skin lesion extended rapidly to the entire right thigh in less than 24 h, and the patient developed septic shock.

**FIGURE 1:**
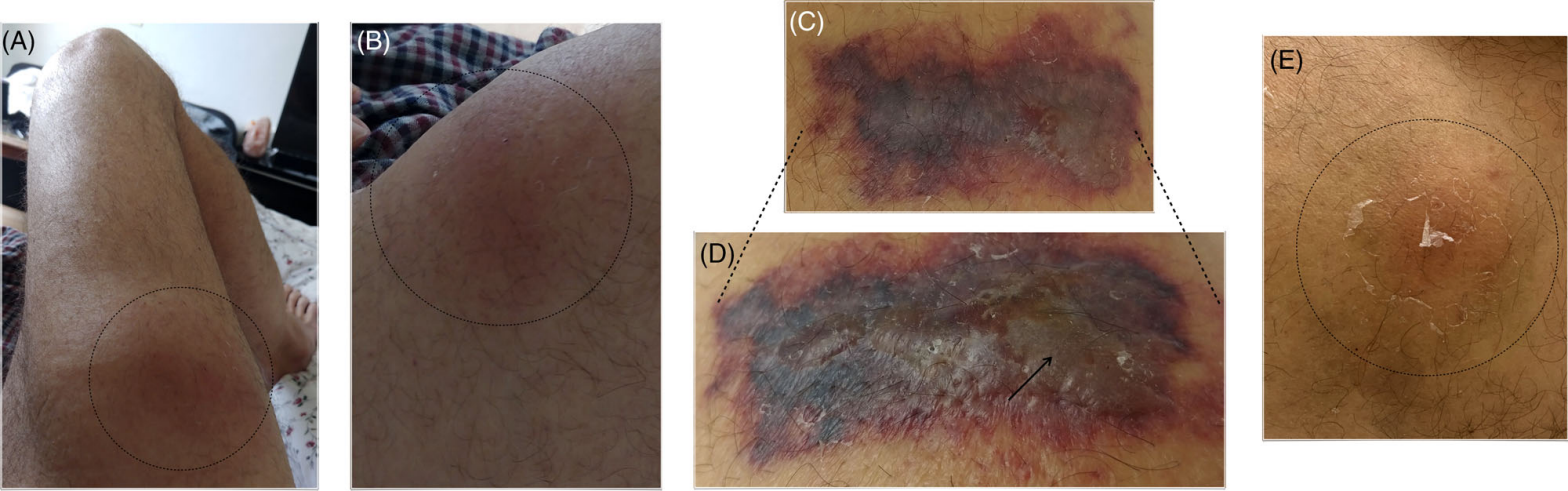
Panel (A): Two days before admitted at hospital. Panel (B): day before admitted at hospital. Panels (C and D): in hospital evaluation. Panel (E): other application subcutaneous azacitidine zone (contralateral leg)

**FIGURE 2:**
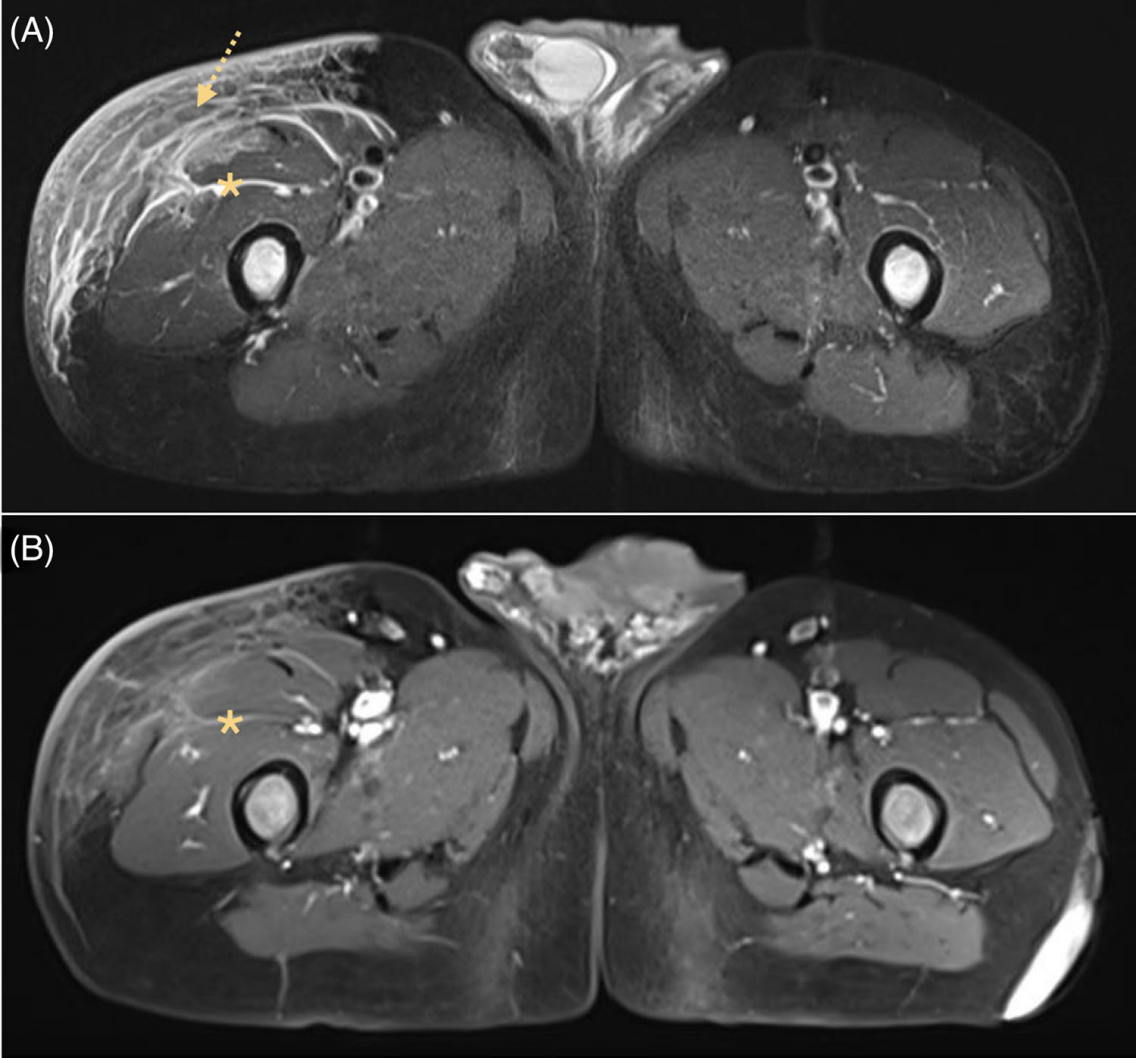
Magnetic resonance imaging (MRI) of upper legs. Signs of fasciitis of the right thigh. Panel (A): short TI inversion recovery (STIR). Increased signal intensity in muscle of the right thigh (*). Marked skin thickening with signal hyperintensity and trabeculation of subcutaneous fat (arrow). Panel (B): fascial enhancement in contrast sequences (*)

He was admitted into the intensive care unit (ICU), and the same day, underwent an emergency fasciotomy and surgical debridement. Wound and blood cultures were continuously negative throughout the process. During his stay in the ICU, several more debridement procedures were indicated, and the patient eventually reached hemodynamic and clinical stability. Unfortunately, after 22 days of hospital stay, the patient passed away due to a ventilator‐associated pneumonia.

Patients with myeloid malignancies may present with a broad range of cutaneous manifestations, including a direct infiltration of tissue by malignant hematopoietic cells or nonspecific lesions (infections, neutrophilic dermatosis, paraneoplastic vasculitis, panniculitis, or cutaneous adverse events by antineoplastic drugs, among others) [1].

Hypomethylating agents, such as azacitidine (analog of cytidine), causes transient demethylation of DNA resulting in cytotoxic effects. The common side effects of azacitidine are cytopenia, general malaise, and gastrointestinal events. Injection site reactions are one of the most common non‐hematological adverse events [2]. Skin events are typically erythema, pruritus, and rash, but skin nodules and neutrophilic dermatosis (Sweet syndrome and pyoderma gangrenosum) are also reported [3]. When azacitidine is injected subcutaneously, it can cause direct damage to the skin cells, such as keratinocytes and endothelial cells, subsequently leading to skin necrosis: Nicolau syndrome (NS). Clinicians should consider this when an injection‐site is ecchymotic and/or develops and erythematous patch, with bulla and intense pain in the immediate post‐injection period [4]. The pathogenesis is not well understood; intra‐arterial or peripheral‐nervous injections might stimulate the sympathetic nerve, causing an acute vasospasm of the vessel, leading to ischemia or inflammation of the vessel structures, followed by thrombosis and necrosis. Various injection administration methods and medications result in NS [4, 5].

NS must be differentiated from NF. The history of a skin lesion secondary to subcutaneous injection with the delayed onset of rapidly progressive local inflammatory changes suggests a closed‐space infection. NF is a rapidly spreading life‐threatening infection of the subcutis and fascia, which could be polymicrobial (type I: e.g., mixed bacteria, including aerobic and anaerobes) or monomicrobial (type II: e.g., hemolytic *streptococci*, methicillin‐resistant *Staphylococcus aureus*). Necrotizing infections can occur after minor breaches of the skin or mucosa or nonpenetrating soft‐tissue injuries in postsurgical and immunocompromised patients [6]. In this case, the skin lesion caused by a subcutaneous puncture (a seemingly trivial injury) was likely the entry point. The rapid clinical deterioration was probably in the context of his condition of immunosuppression due to senescence and MDS. To our knowledge, there are only three published case reports of patients with MDS in which NF occurred after injections of azacitidine, but only in one of them subcutaneously [7, 8]. It is likely that in these cases, the initial mechanism was the same as in NS: vasospasm, ischemia, necrosis, and, subsequently, the development of a deep superinfection (NF).

NF should be considered in patients with intense local pain (in crescendo), erythema of the skin that later becomes purplish or vesiculated (Figure 1, panels C,D), wooden‐hard induration of the subcutaneous tissues and systemic manifestations of sepsis [6, 9]. The process progresses rapidly over several days. Within 3–5 days after onset, skin breakdown with bullae and frank cutaneous gangrene can be seen as was presented in our patient; the speed of the evolution is a limitation, given that, the local reactions typical of the drug can mask a deep necrotizing infection.

Absence of fever, cutaneous manifestations, and/or findings on imaging tests (e.g., gas in the deep tissue) are some of the pitfalls in the diagnosis of NF. The presence of crepitus or imaging evidence of gas in the tissues should prompt immediate surgical consultation [6]. MRI may show thickening and hyperintensity of intermuscular fascia on T2‐weighted images, findings that are sensitive but not entirely specific [9].

Blood cultures are positive in ∼60% of patients with monomicrobial NF and only in 20% of polymicrobial NF. Gram's staining and culture of surgically obtained material is crucial for guiding treatment [9, 10, 11]. In our patient, blood cultures and tissue samples were consistently negative considering that he was already on antibiotic therapy. This highlights the importance of having a low threshold for diagnosis and promptly starting broad‐spectrum antibiotic coverage. The Infectious Diseases Society of America [9] recommends vancomycin or linezolid plus one of the following therapies: piperacillin–tazobactam, carbapenem, or ceftriaxone–metronidazole. Other therapies include intravenous immune globulin, but its effectiveness is controversial [11]. For patients with clinical diagnosis of NF, prompt surgical exploration is extremely important [6].

NF is associated with considerable mortality, even with optimal therapy (ranges between 14% and 34%) [6, 9, 11]. In this case, nosocomial infectious complications led to a fatal outcome. In the other three reported cases, two had a fatal outcome because of poor infection control [7, 8].

In summary, clinical physicians should be aware and have a high suspicious threshold. Expedited diagnosis, prompt surgical intervention, and appropriate antibiotic treatment are essential to limit complications.

## AUTHOR CONTRIBUTIONS

All authors had access to the data and a role in writing this manuscript.

## CONFLICT OF INTEREST

The authors declare they have no conflicts of interest.

## FUNDING INFORMATION

The authors received no specific funding for this work.

2

## Data Availability

The information will be provided if requested in a particular way.
